# Items, Selections and Remarks

**Published:** 1870-06

**Authors:** W. W. Miner


### Items, Selections and Remarks.

BY W. W. MINER, A. B.

M. Behier, knowing of the antagonism of morphia and atropia, which was described by Von Graefe in 1862, gave seventy-five drops of laudanum in doses of ten drops every ten minutes, to a patient who had taken one-fifth of a grain of sulphate of atropia, and with the effect of diminishing the intensity of the toxic symptoms, whereas symptoms of poisoning by opium would have been produced had not atropia previously been taken.——Dr. H. C. May, of Corning, N. Y., stated in the *Record* for June 1st, a case of atropine versus opium, and says that he is firmly persuaded that but for the antidotal effects of the atropine, the patient would have succumbed to the narcotic effects of the morphine. ——The use of an atomized solution of lactic acid, as well as of lime-water spray, has been recommended for the removal of the fibrinous exudation of croup.——Dr. Geo. F. Jenkins, of Sandusky, Iowa, published in the *Philadelphia Reporter*, the history of a severe case of rattlesnake poisoning which he treated by the hypodermic injection into the wound of one drachm of aqua ammonia, and again of the same amount of ammonia diluted with an equal amount of water, inserted at a point near the wound; whisky was also made use of. He says that he firmly believes that the ammonia saved the patient's life, and were he called to another case, especially if it were soon after the wound was inflicted, he would not hesitate to rely exclusively upon the hypodermic injection of aqua ammonia.——In the furor caused by the great prevalence of small pox in Paris at the present time, two thousand persons were vaccinated at one of the establishments where virus, directly from kine, was made use of. The experience of the Paris physician within the last few weeks, has abundantly confirmed belief in the value of vaccination as a protection against the ravages of small pox, and has also not confirmed belief in the superior virtue of virus from the kine.

Dr. Charles Daremberg has been elected to the newly vacated chair of History of Medicine, in the Faculty of the Paris School of Medicine. Drs. Lorain, Bouchet, Maurice, and Raymond, were his competitors for the position. Dr. Daremberg has been engaged for many years in writing a "History of the Medical Sciences," the last volume of which has just been issued by Baillière & Son. The *New York Medical Journal* speaks of the work as being very erudite and interesting, and recommends it to the Sydenham Society for translation.——Dr. Christopher Johnson, of Baltimore, has been appointed to the chair of Surgery in the University of Maryland, which position Dr. Nathan R. Smith has lately resigned.——Dr. Albert Day has retired from the Superintendency of the State Inebriate Asylum at Binghamton, and has established in Massachusetts a private institution where he can employ, without limitation, the methods he believes to be most efficacious in the treatment of patients under his care. Dr. Daniel G. Dodge is his successor in the Asylum at Binghampton. ——Dr. Henry Gibbons, Sr., has been elected President, and Dr. T. M. Logan, permanent Secretary, of the California State Board of Health.——The annual meeting of the Maine State Medical Society will be held at Bangor, commencing Tuesday, June 28th, and continuing three days.——The Society for the Protection of Infants held an enthusiastic meeting at Paris this year, at which Dr. Boudet was elected President, and Dr. Mayer, Secretary.

R. J. Fowler writes from Paris to the *Druggists' Circular*, a description of the "Lelanche Battery," now extensively used in France. It consists of an outer jar containing a concentrated solution of sal ammoniac, and a sheet or rod of amalgamated zinc; the solution should only half fill the jar, and in it should be placed a porous jar containing bruised peroxide of manganese, in which is immersed a rod of good gas carbon. The battery uses up only while its circuit is closed, and twenty-eight cells of the Lelanche battery are said to be equal to forty of Daniel's. The amount of chemical action in this battery, and the length of its duration is said to be exactly proportionate to the work it is called upon to perform.

The *Dental Cosmos* states a case that promises to be fatal, of a dentist resident in a small town in the State of New York, who, in operating for a patient suffering from a syphilitic difficulty, contracted the disease from absorption of the virus at a point on one of his fingers where the skin was broken by a hangnail. The dentist, not recognizing the disease, was soon forced to leave his practice; and now, is not expected to long survive the severe effects of the disease.——Dr. Turner, in his "Second Annual Report of the State Inebriate Asylum," states that out of 1406 cases of delirium tremens which have come under his observation, 980 had an inebriate parent or grand parent, or both. He believes if the history of each patient's ancestors were known, it would be found that 8 out of 10 of them were free users of alcoholic drinks.—*Medical Record.*——A monument to Dr. Horace Wells, the discoverer of anæsthesia, is shortly to be erected in Hartford, Conn.——Fifty-three thousand infants are annually born in Paris, of which twelve to thirteen thousand at the end of the year are dead.——Dr. Beigel exhibited before the London Pathological Society, April 5th, the pubic parts taken from the body of a member of a religious sect in Russia, known under the name of the sceptics. The penis, scrotum, and testicles, are removed with one sweep, and the specimen was the first that had been obtained in Russia, so successful had the sect been in preserving secrecy. He also exhibited photographs of members of the sect, showing the change in physiognomy following the removal of the genitals.—*Record.*

Dr. Benjamin Howard has been awarded the prize this year by the American Medical Association for his essay on "Aneurism."——The Alumni Association of the College of Physicians and Surgeons of New York City, has given this year's prize to Dr. Andrew H. Smith for an essay upon "Oxygen Gas as a Remedy in Diseases."——Seventeen ladies have entered the London University examinations this year.——One hundred and fifteen persons died with cerebro-spinal meningitis in Coffee county, Ga., in the month of March.——It is said that man requires one twenty-third of his weight in food daily.——Clinics, in mental diseases, have been given in Great Britain at the University of Cambridge, by Dr. McKenzie Bacon; at Guy's Hospital, by Dr. Thomas Dickson; and at Glasgow, by Dr. Charles Robertson.——The June number of the *Gynæcological Journal* appears in full mourning in honor to the memory of the late Prof. James Y. Simpson.——The recent troubles at the Paris School of Medicine between the students and M. Tardieu, are at an end.——A lady graduate in medicine has obtained from the *New York Medical Gazette*, a prize for the best clinical reports presented to it for publication.

Dr. Mathews Duncan is thought of as successor of the late Sir James Y. Simpson in the University of Edinburgh.——Dr. Paul F. Eve has accepted the chair of Clinical and Operative Surgery in the University of Nashville.——

Dr. Hering, of Vienna, succeeds the late Prof. Purkinje in the chair of Physiology at Prague. Prof. Helmholtz, of Heidelberg, declined leaving his present position.——Sir Thomas Watson, who has been suffering with ——, is now recovering.——Rokitansky has been elected President of the Imperial Academy of Science at Vienna.——Prof. C. L. Ford, of Long Island College Hospital, Brooklyn, N. Y., embarked upon the "Marathon" for a trip to Europe, whence he returns in season for the commencement of the next lecture course.

